# Theoretical generalization of normal and sick coronary arteries with fractal dimensions and the arterial intrinsic mathematical harmony

**DOI:** 10.1186/1756-6649-10-1

**Published:** 2010-09-17

**Authors:** Javier O Rodríguez, Signed E Prieto, Catalina Correa, Pedro A Bernal, Germán E Puerta, Sarith Vitery, Yolanda Soracipa, Diana Muñoz

**Affiliations:** 1 MD. Insight Group Director - Investigation Center, Clínica del Country. Professor and Director of the research area: "Mathematical and Physical Theories Applied to Medicine". Universidad Militar Nueva Granada, Cr 11 No. 101-80, Bogotá, Colombia; 2 Insight Group researcher - Investigation Center, Clínica del Country. Bogotá, Colombia; 3 Psychologist. Insight Group researcher- Investigation Center, Clínica del Country. Bogotá, Colombia; 4 Systems Engineering Student. Universidad Nacional de Colombia. Insight Group researcher - Investigation Center, Clínica del Country. Bogotá, Colombia; 5 Medicine student. Universidad Militar Nueva Granada, Bogotá, Colombia; 6 Degree in Physics student. Universidad Pedagógica Nacional. Insight Group researcher- Investigation Center, Clínica del Country. Bogotá, Colombia; 7 Medicine student. Universidad Militar Nueva Granada, Bogotá, Colombia. Special Internship: "Mathematical and Physical Theories Applied to Medicine

## Abstract

**Background:**

Fractal geometry is employ to characterize the irregular objects and had been used in experimental and clinic applications. Starting from a previous work, here we made a theoretical research based on a geometric generalization of the experimental results, to develop a theoretical generalization of the stenotic and restenotic process, based on fractal geometry and Intrinsic Mathematical Harmony.

**Methods:**

Starting from all the possibilities of space occupation in box-counting space, all arterial prototypes differentiating normality and disease were obtained with a computational simulation. Measures from 2 normal and 3 re-stenosed arteries were used as spatial limits of the generalization.

**Results:**

A new methodology in animal experimentation was developed, based on fractal geometric generalization. With this methodology, it was founded that the occupation space possibilities in the stenotic process are finite and that 69,249 arterial prototypes are obtained as a total.

**Conclusions:**

The Intrinsic Mathematical Harmony reveals a supra-molecular geometric self-organization, where the finite and discrete fractal dimensions of arterial layers evaluate objectively the arterial stenosis and restenosis process.

## Background

The fractal geometry, developed by Benoit Mandelbrot, allows irregular objects characterization, through fractal dimensions [1,2]. There are several fractal dimension definitions and different methodologies for calculation, applied according to measured object [2]. For wild fractals, such as those that characterize the morphology, box counting method is used. Fractal geometry have been used in experimental and clinical applications [3,4], for example to differentiate cardiac lesion degree in angiography, to characterize pre-neoplasic cervical cells or normal and abnormal erythrocyte morphology [5-7]. However, in some cases, isolated fractal measures not differentiate normality and disease, and could present limitations for their effective application [8].

This problem was analyzed [9] in the restenosis phenomena. Although arteries present irregular form and structure, it is common to do Euclidean measurements of their shape for their posterior statistical analysis [10,11]. Rodríguez et al. [9] demonstrated that isolated fractal dimensions of whole artery or parts can not differentiate between groups with and without restenosis, and developed a new methodology for differentiation based on Intrinsic Mathematical Harmony (IMH), which allows to compare the fractal dimensions of parts and whole artery. This concept is mathematically defined as the similarity degree or difference between units and first three decimal ciphers - known as significant ciphers-, to compare fractal dimensions of parts and totality of artery. Fractal dimension measures of objects components of healthy arteries were similar to whole artery measure, showing differences in third significant cipher. For example, artery number 17 presented fractal dimensions equals to 1.0565 and 1.0524 on island 1 and island 2, compared to measure of whole artery that was equals to 1.0544. In the other arteries, treated with octreotide or placebo, the difference between parts and whole artery was evident starting from the first significant cipher, and even in unit, as in the artery number 10, which presented values equals to 1.0458, 0.9643 and 1.1699 in the island 1, island 2 and the whole artery respectively. These values compared by the IMH determined the difference between healthy and diseased arteries, with values of 3, 3 and 3 for artery 17, while artery 10 presents values equals to 0, 0 and 1.

The Intrinsic Mathematical Harmony concept was adapted and used for the differentiation between ventricles with ejection fraction less than 40% compared to normal ones. Rodríguez et al. [6] assessed the degree of similarity between the fractal dimensions of the left ventricular contours in cardiac dynamics in systole, diastole and the whole object. The degree of similarity between the fractal dimensions of the comparisons made in the contours of a healthy ventricle varies between 2^0.9 ^and 2^10^, while those with a ventricular ejection fraction less than 40% are between 2^10 ^and 2^500 ^at least in one of the comparisons made, successfully distinguishing normal of severe cases with mathematical, objective and reproducible measures.

The purpose of this investigation is to develop a generalization of arterial fractal geometric structure evaluation, taking as fundament the Intrinsic Mathematical Harmony concept, through of a software and in this way obtain the finite set of possible normal and sick arteries, designed as prototypes.

## Methods

In order to do this investigation, fundamentally mathematical, coronary arteries images and their fractal dimensions, previously obtained from the arterial structural characterization study developed by Rodríguez et al. (2002) at Fundación Cardio Infantil were used. Two normal and three sick arteries evaluated with Intrinsic Mathematical Harmony concept were chosen for generalization development.

The fractal dimension was calculated using only two grids, in order to make a calculus simplification like in previous research used as reference [9]. Two squaregrids were built, in which squares side of one of them, is double of squares side of the other one. Then each mentioned square-grids were superposed over the images, in order to do square account required for box-counting method application [2].

The number of normal prototypes was calculated following the IMH established definition, where at least first decimal cipher of fractal dimension for component parts and totality must be equal, for normal arteries. The 17 arteries from the previous study [9] which fractal dimensions are equal until the second cipher were taken as initial prototype. The fact that Box-counting fractal dimension is defined in interval(0-2) was also used. In this way, calculation of normal prototypes was done starting from the case in which three measured regions have a fractal dimension where two first significant ciphers and unit are zero, adding 0.01 consecutively to obtain successively all possible values for fractal dimension, for parts and totality simultaneously.

**Figure 1:**
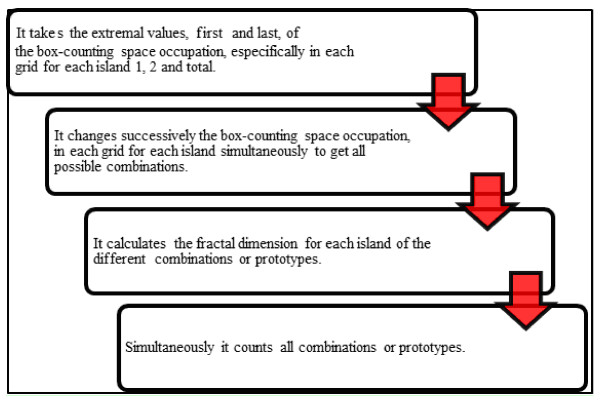
**Flow chart of the functions performed by the developed software**.

Based on IMH, a software in C++ language was designed, capable to simulate arterial deformation. This software allows to get all the possible arterial prototypes in occlusion process that correspond to stenosed or restenosed arteries, where each possible combination constitutes an arterial prototype, obtaining all geometric possibilities of box-counting space occupation by arterial layers and for each specific artery, including all the possibilities of experimental vascular remodeling (see figure 1).

**Figure 2:**
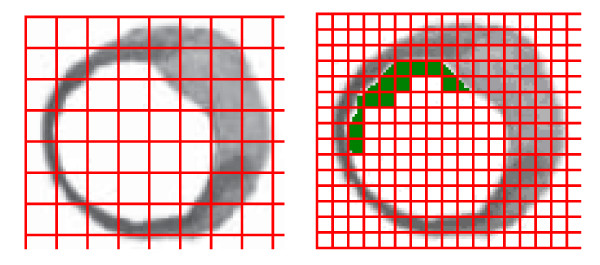
**Island 1 with the two superposed Box-Counting grids**. The green area on the right image is an example of the theoretical remodeling of this island, obtained with the developed software.

**Figure 3:**
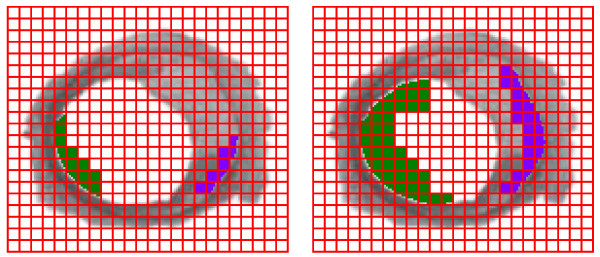
**Example of two arterial prototypes theoretically obtained using the developed software**. The green area corresponds to the remodeling simulation of the island 1, the blue zone corresponds to the remodeling simulation of the island 2. The left image corresponds to a lower level of occlusion, with respect to the right one.

According to the used methodology, each arterial layer is described by a set of occupied squares in each of the grids (see figure 2). The maximum size of islands occupation corresponds to the maximum number of spaces occupied by the islands in the studied prototypes. Different sets of occupied squares describe different arterial prototypes (As an example see figure 3). Considering the possible sets of squares marked out by all the possible arterial contours and doing all the possible combinations, all possible arterial prototypes are obtained. The fractal dimension is calculated considering the number of squares occupied by the object evaluated, it can take real values in the range between 0 and 2, and its value changes according to the variation of the number of squares occupied by the object.

Finally, with the same software fractal dimensions were calculated and all possible sick arteries prototypes were counted, including all experimental vascular remodeling possibilities. In this work, islands represent arterial layers histologically differentiated. When external or internal elastic lamina breaks up, the minimum union way between the two extremes is taken into account for calculus. In this way, the generalization includes all lesion grades, without take into account if laminas are broken or not.

### Mathematical Analysis

The theoretically calculated fractal dimensions were compared to those experimentally obtained [9] to confirm correspondence with the developed generalization. Due that the software simulates arterial deformation, it leads to a unique set of possible arterial structures in a general and complete way, without requiring repetitions of experiment; avoiding statistical analysis and grate samples studies to prove the correspondence with any particular artery.

Some cases on which fractal dimension values were zero were not taken into account in results, because it does not joint to any arterial prototype, showing in this way that not all the mathematical possibilities have experimental sense.

### Definitions

**Fractal: **From the Latin fractus, it means irregularity used as substantive or irregular as an adjective.

**Fractal Dimension: **numerical measurement to characterize irregularity degree. The fractal dimension definition used in this case is Box-Counting fractal dimension [2].

**Artery's Intrinsic Mathematical Harmony (IMH) **[9]: Similarity degree or difference between units and significant ciphers of fractal dimensions of island's parts, with artery totality.

**Arterial Fractal prototype: **Geometric combination of simultaneous occupation of Box-counting space by different constitutive regions, islands, and totality of arterial structure, which fractal dimensions correspond to some particular artery evaluated with IMH. (Definition done by the first author.)

**Island: **Fractal object defined starting from limits of selected arterial layers [9].

## Results

At the execution of the software and the fractal dimensions calculus for the three defined regions from sick arteries, it was found that fractal dimension of island 1 can take values between 0.0443 and 1.5670; Island 2, between 0.7520 and 1.3168 and Total Island, between 0.0395 and 1.6147. The interval of fractal dimensions from Total Island is bigger than the other islands. The interval of values for fractal dimensions of island 1 was bigger than the island 2.

The arterial prototypes that correspond to all possible sick arteries are 69 049, no matter if those are associated to stenosis or restenosis, while 200 prototypes of healthy arteries were obtained, starting from the difference between healthy and unhealthy arteries based on IMH concept. So, considering every possible normal and sick arteries prototypes, there are 69 249 in total. This result shows how can a normal artery evolves into a sick one, without significance of the cutting place. Some data obtained in simulation is showed in tables 1 and 2.

**Table 1:** Fractal dimensions of three normal prototypes obtained from the normal arteries simulation based on IMH.

	Island 1	Island 2	Total island
1	0.8715	0.8726	0.8737
2	0.8434*	0.8479*	0.8930*
3	1.0565*	1.0524*	1.0544*

The numbers with an asterisk are fractal dimensions present in empirical arteries from the previous study [9].

**Table 2:** Fractal dimensions of ten sick prototypes obtained from the simulation.

	Island 1	Island 2	Total island
1	0.9368	0.9643	1.0604
2	1.0641	1.0494	1.0780
3	0.9328	1.0931	1.0199
4	0.7629	0.8883	0.7608
5	0.8826	0.9845	0.7776
6	1.0230	1.1868	1.0824
7	0.9765	0.8771	0.9349
8	1.2016	1.1015	1.2854
9	0.9510	1.0435	0.8382
10	0.9259*	1.143*	1.2094*

The numbers with an asterisk are fractal dimensions present in empirical arteries from the previous study [9].

The experimental measures already obtained [9] are included at theoretical calculus (see table 3); some examples of them are shown in table 2.

**Table 3:** Extreme values of the fractal dimensions of theoretical and experimental sick arteries.

	Theoretical superior value	Experimental superior value	Experimental inferior value	Theoretical inferior value
Island 1	1.5670	1.0919	0.8073	0.0443
Island 2	1.3166	1.0809	0.8821	0.7520
Total Island	1.6147	1.3599	0.9068	0.0395

The experimental values are a previous result [9].

## Discussion

This is the first work in which a mathematical generalization is constructed starting from fractal dimensions calculus and IMH concept, based on a new theoretical-practical investigation methodology in animal experimentation area. With this methodology, every possible fractal prototypes of normal and sick arteries were calculated, finding a finite quantity. The numeric limits used can change without affecting the geometric characterization. This methodology does not require descriptive classifications of stenosis or restenosis grades.

The Standard methodology to evaluate arterial occlusion proposed by some investigators [12], evaluates the response to barotrauma starting from revascularization indexes defined from Euclidean geometry. Based on a simple theoretical-practical experiment that takes into account irregularity of arterial layers and whole artery in box-counting space, it was anticipated that total number of arteries prototypes is finite, showing fractal geometric auto-organization of artery, with experimental confirmations [9] founded as particular cases inside the totality of calculated prototypes. The initial prototypes used in simulation were selected because they are prototypes of normality and advanced restenosis without diagnostics doubts, whose geometric characteristics allow developing an induction process that includes all arteries that are contained within these geometric ranges.

In a previous work [13], it was developed a geometric characterization of red blood cells morphology that improves the conventional clinical diagnosis of blood samples, differentiating normal and diseased samples by means of Euclidean and fractal geometries. These parameters are applicable to transfusion bags and pre or post transfusion evaluations. The results founded in this work could be refined in the future using fractal and Euclidean measures together, as in the cited work.

Multiple researches in medicine based on physics and mathematics theories have been developed, based on a-causality of theoretical physics and geometry. Among these some characterizations that allow an objective mathematical differentiation for binding and not binding peptides to red blood cell receptor, based on sets theory and with probability and entropy theories too [14,15]. The binding phenomenon of nonameric peptides to HLA class II was also characterized, based on set theory, work that was useful as basis to develop a binding theory to HLA class II based on probability, combinatory and entropy theories, which predicts the binding and not binding state of 161 peptides tested with 100% success [16,17]. In cardiac morphology and dynamical field, a diagnostic methodology for clinical application of fetal heart rate monitoring was developed, based on dynamical systems theory and Zipf-Mandelbrot law, which was refined and presented at XVIII FIGO Congress [18]. A methodology of adults cardiac dynamic evaluation in Holter, based on the theory of dynamical systems was also developed, that differentiate acute dynamics from chronic and normal ones [19]. The work in which concept of IMH was developed, and basis of this work, follows same essential methodology that mentioned works, using geometric characterizations and searching for acausal mathematical relations. In words of Prigogine, we only have temporal windows to comprehend the reality [20]; in this case those are the simultaneous mathematical relations of fractal dimensions, which constitute prototypes, where experimentally measured arteries are inside of the theoretically calculated prototypes totality.

In this methodology only with the determination of harmonic relation between parts and whole object is possible to know the occupied space by an irregular object. This shows the simplicity of complexity and that it is possible to create a methodology able to find the subjacent order into irregularity. This kind of methodology can be used in human body studies and in experimental models with animals, being able to obtain results with small samples, regardless statistical and epidemiological studies [11].

## Limitations

The developed generalization shows all the possibilities of occupation of a defined fractal space, but it doesn't establish specific artery thicknesses or longitudes.

## Conclusions

A new methodology of scientific investigation in animal experimentation was developed, based on IMH in fractal space of Box-counting, that allows a geometric and numeric objective generalization, capable of simulate variability and complexity of coronary stenosis and restenosis, without take into account experimental classifications.

From this new methodology perspective, arterial stenosis and restenosis are revealed as a fractal supra-molecular geometric auto-organization phenomenon.

Based on the developed methodology, a finite quantity of 200 normal and 69049 sick arterial prototypes are obtained, calculating 69 249 in total.

This methodology allows getting objective and precise mathematical results with simple experiments, without a great sample, avoiding the unnecessary death of animals, and additionally allowing an optimum use of financial sources and time.

## Competing interests

The authors declare that they have no competing interests.

## Authors' contributions

JOR conceived the study, and participated in its design and coordination, SEP drafted the document, participated in data analysis, CC drafted the document, participated in the design of the study and data analysis, PAB developed software and mathematical - fractal calculations and drafted the document, GEP participated in data recollection and its systematization, SV participated in data recollection and its systematization, YS drafted the document and participated in mathematical calculations, DM drafted the document and participated in mathematical calculations.

All authors read and approved the final manuscript.

## Pre-publication history

The pre-publication history for this paper can be accessed here:

http://www.biomedcentral.com/1756-6649/10/1/prepub
